# The Impact of Heat Stress on Dairy Cattle: Effects on Milk Quality, Rumination Behaviour, and Reticulorumen pH Response Using Machine Learning Models

**DOI:** 10.3390/bios15090608

**Published:** 2025-09-15

**Authors:** Karina Džermeikaitė, Justina Krištolaitytė, Dovilė Malašauskienė, Samanta Arlauskaitė, Akvilė Girdauskaitė, Ramūnas Antanaitis

**Affiliations:** Animal Clinic, Veterinary Academy, Lithuania University of Health Sciences, Tilžės Str. 18, LT-47181 Kaunas, Lithuania; justina.kristolaityte@lsmu.lt (J.K.); dovile.malasauskiene@lsmu.lt (D.M.); samanta.arlauskaite@lsmu.lt (S.A.); akvile.girdauskaite@lsmu.lt (A.G.); ramunas.antanaitis@lsmu.lt (R.A.)

**Keywords:** dairy cows, artificial intelligence, biosensors, precision livestock farming, temperature–humidity index, early detection

## Abstract

Heat stress has a major impact on dairy cow health and productivity, especially during early lactation. Conventional heat stress monitoring methods frequently rely on single indicators, such as the temperature–humidity index (THI), which may miss subtle physiological and metabolic responses. This study presents a novel threshold-based classification framework that integrates biologically meaningful combinations of environmental, behavioural, and physiological variables to detect early-stage heat stress responses in dairy cows. Six composite heat stress conditions (C1–C6) were developed using real-time THI, milk temperature, reticulorumen pH, rumination time, milk lactose, and milk fat-to-protein ratio. The study applied and assessed five supervised machine learning models (Partial Least Squares Discriminant Analysis (PLS-DA), Support Vector Machine (SVM), Random Forest (RF0, Neural Network (NN), and an Ensemble approach) trained on daily datasets gathered from early-lactation dairy cows fitted with intraruminal boluses and monitored through milking parlour sensor systems. The dataset comprised approximately 36,000 matched records from 200 cows monitored over 60 days. The highest classification performance was observed for RF and NN models, particularly under C1 (THI > 73 and milk temperature > 38.6 °C) and C6 (THI > 74 and milk temperature > 38.7 °C), with AUC values exceeding 0.90. SHAP analysis revealed that milk temperature, THI, rumination time, and milk lactose were the most informative features across conditions. This integrative approach enhances precision livestock monitoring by enabling individualised heat stress risk classification well before clinical or production-level consequences emerge.

## 1. Introduction

Climate change presents a global dilemma that profoundly affects livestock, with heat stress becoming a pivotal concern in dairy farming. [1]. Dairy cows are especially susceptible to variations in external temperature because of their elevated metabolic heat output and restricted ability to dissipate heat [2,3,4]. Approximately 60% of dairy farms worldwide are located in heat-stress regions [5], where seasonal declines in milk production and compositional changes due to high ambient temperatures remain persistent concerns for dairy producers [3,6,7].

According to Selye’s stress theory, heat-stressed animals exhibit a nonspecific immune response that negatively affects their productive and physiological characteristics [8]. Such temperature and humidity conditions are usually quantified by a compound index called the temperature–humidity index (THI) [9]. THI values exceeding 72 are considered detrimental to dairy cows, leading to decreased feed intake, compromised milk quality, and increased susceptibility to disease [10,11]. Lactating cows will start to suffer from the detrimental effects of heat stress when the THI surpasses 68 [12,13]. However, heat stress not only leads to a reduction in milk yield but also alters milk composition, affecting protein, fat, solid non-fat, casein, and lactose contents, as well as milk urea and somatic cell score (SCC), while also contributing to elevated SCC and fluctuations in milk fat and protein concentrations [14]. The most significant impacts of heat stress on milk composition generally manifest within the initial 60 days of lactation [15].

In parallel, machine learning (ML) approaches have shown promise for improving disease detection and physiological state classification in dairy herds [16,17,18]. ML may significantly enhance the analysis of behaviour [19]. In contrast to conventional multivariate regression, ML models are superior in identifying intricate non-linear interactions among variables [20]. By integrating diverse data streams (e.g., environmental, behavioural, and milk-related variables), ML models can enhance the sensitivity and specificity of early warning systems. Nevertheless, limited research has systematically assessed the efficacy of diverse ML algorithms in identifying physiological and metabolic instability associated with heat stress in dairy cows through integrated biosensor data [12,21,22]. Nonetheless, limits persist in the utilisation of machine learning techniques in this domain, chiefly attributable to data availability constraints, resulting in insufficient exploration of critical predictive elements [20].

Unlike prior studies that typically evaluate one variable at a time (e.g., THI alone or milk yield decline) [23,24,25,26], this study proposes a multifactorial classification logic grounded in animal physiology and refined using actual herd-level data [27,28,29]. To our knowledge, this is the first study to formalise threshold-based binary classifications across multiple physiological axes (e.g., reticulorumen pH, milk fat-to-protein ratio (FPR), lactose, rumination time, milk temperature) and apply them as supervised learning targets using high-resolution farm data during early lactation. Unlike previous approaches that rely on population-level daily summaries or static heat stress indices [1,30,31], our method leverages fine-resolution, real-time data (e.g., rumination, milk temperature, reticulorumen pH) and integrates them with environmental metrics such as THI. The proposed approach addresses a critical gap in current livestock heat stress monitoring systems, which often fail to identify subclinical or behaviourally mediated stress responses prior to production losses.

Rather than relying on isolated variables or generalised heat stress cut-offs, we designed compound thresholds based on (i) existing literature on heat stress pathophysiology in dairy cows, (ii) known biomarker thresholds associated with subclinical disorders (e.g., ruminal acidosis, metabolic load), and (iii) empirical data distribution from our cohort collected during early lactation (first 60 days postpartum). Although these threshold rules are not yet industry-standard, their construction follows a mechanistic rationale supported by current dairy physiology and heat stress literature. Importantly, they serve as testable hypotheses for evaluating early and subclinical responses to heat load. The combination of these thresholds with contextual physiological and behavioural indicators, such as reduced rumination or low reticulorumen pH, allows for a composite view of heat- and metabolism-related stress responses. By grounding each threshold in both prior evidence and internal data structure, we ensured that C1–C6 reflect biologically plausible and statistically testable conditions rather than arbitrary classifications.

We hypothesise that compound threshold conditions, incorporating both thermal (e.g., THI, milk temperature) and metabolic/behavioural (e.g., milk lactose, reticulorumen pH, FPR, rumination time) indicators, can be used to detect early physiological and lactational alterations in dairy cows exposed to heat stress. These thresholds are designed to reflect biologically plausible stress states before clinical signs become evident.

This study aimed to achieve two objectives: (1) to assess the physiological, behavioural, and milk quality responses of early lactation dairy cows subjected to six threshold-based heat stress conditions (C1–C6), and (2) to evaluate the classification performance and interpretability of five supervised machine learning algorithms—Support Vector Machine (SVM), Partial Least Squares Discriminant Analysis (PLS-DA), Random Forest (RF), Neural Network (NN), and an Ensemble model—applied to these conditions. Through this approach, we aimed to identify sensitive and biologically meaningful indicators of heat stress and assess their potential for early prediction and decision support in precision dairy systems.

## 2. Materials and Methods

This study was conducted in accordance with the Lithuanian Law on Animal Welfare and Protection, under approval number G2-227. The experiment took place between 1 June 2024, and 31 August 2024, on a Lithuanian dairy farm that houses 1500 lactating cows in free-stall barns. The farm, located at latitude 54.97378759003201 and longitude 23.76954146935687, utilises a DeLaval milking system (DeLaval Inc., Tumba, Sweden) to milk 1000 cows twice daily. In order to guarantee optimal housing conditions, the stables are outfitted with ventilation systems (DeLaval Inc., Tumba, Sweden). Each day, milking was conducted in a parlour system at 5 a.m., 1 p.m., and 5 p.m.

For the study, 200 lactating cows were selected from a group of 1000 clinically examined animals. Only cows in their second or later lactations, within 5 to 10 days post-calving, were included. Only clinically healthy cows were included in the study, based on a comprehensive health assessment. Each cow underwent a detailed physical examination to verify overall well-being and rule out any systemic illnesses or debilitating conditions. The evaluation entailed the observation of general appearance and behaviour for potential indications of disease. Cows exhibiting symptoms of systemic illness were excluded in order to minimise variability and guarantee data reliability. The clinical evaluations verified that all of the selected cows were in excellent health, with no signs of clinical disorders.

The farm utilised a computerised record-keeping system (Delpro, DeLaval Inc., Tumba, Sweden) to extract and arrange essential data, including breed, lactation number, last calving date, and milk yield, into a spreadsheet. The days in milk for each cow were obtained by calculating the interval between the last calving date and the initiation of data collection.

The cows were provided with a total mixed ration (TMR) meticulously formulated to meet their nutritional and physiological needs. This persisted throughout the investigation. The animals had constant access to fresh water and were fed bi-daily at 6 a.m. and 6 p.m. The diet was meticulously crafted by a nutritionist to guarantee that it satisfied all of the essential nutrient requirements for optimal health and milk production. The remaining feed was removed on a daily basis at 5 a.m. and 5 p.m. The nutritional composition of the diet is illustrated in Table 1. The diet included a high-energy grain concentrate, composed of 50% barley and 50% wheat, providing a readily fermentable carbohydrate source to support lactation performance and optimise rumen function. The diet provided to the cows is shown in Table 2. This nutritional formulation was specifically designed to meet the energy and dietary needs of Holstein cows weighing between 550 and 650 kg. 12,500 kilogrammes of energy-corrected milk produced by each cow during each lactation, with a fat content of 4.2% and a protein content of 3.6% on average. The TMR was prepared in alignment with the nutritional requirements of dairy cattle (NRCs) to ensure that Lithuanian Holstein dairy cows received a well-balanced diet that supported their physiological demands and optimal productivity [32].

The TMR provided to the cows is shown in Table 1.

### 2.1. Collected Variables

All registered parameters were recorded from 1 June 2024, to 31 August 2024. Cows were specifically selected during the 5–10-day postpartum period, with data recorded from 1 June 2024, to 31 August 2024, based on our previous study [33]. Environmental heat stressors and physiological responses were recorded every 10 min. The experimental trial was conducted over a period of 60 days after calving. This selection was informed by prior research, which identified early lactation as a highly sensitive phase for dairy cows, characterised by intense metabolic adaptations, immune challenges, and pronounced physiological stress responses [34]. In this study, milk composition parameters, including milk yield, fat, protein, FPR, lactose, and temperature, were assessed using the BROLIS HerdLine in-line milk analyser (Brolis Sensor Technology, Vilnius, Lithuania). Additionally, various behavioural and physiological parameters of the cows were monitored through SmaXtec boluses (SmaXtec Animal Care GmbH, Graz, Austria), which recorded rumination time (min/day), body temperature (°C), reticulorumen pH, water intake (L/day), and activity levels (h/day). Additionally, environmental conditions on the farm were continuously recorded using a SmaXtec climate sensor, which measured the THI (SmaXtec Animal Care GmbH, Graz, Austria).

### 2.2. Milk Parameters

In this study, milk composition was continuously monitored using the BROLIS HerdLine in-line milk analyser. This advanced system allowed for real-time measurement of lactose, milk temperature, milk fat, protein content, and the FPR from each sample collected during milking. Milk composition indicators were recorded three times daily during milking at 5 a.m., 1 p.m., and 5 p.m. The device operates using a GaSb broadly tunable external cavity laser-based spectrometer, functioning within the 2100–2400 nm spectral range. By employing transmission-mode monitoring, the analyser continuously tracks milk flow throughout the milking process, enabling precise individual measurements. Through the analysis of molecular absorption spectra, this technology effectively serves as an on-farm miniature spectroscopic laboratory, facilitating the accurate determination of key milk components. The compact design allows for seamless integration into the milking parlour, where it is directly attached to the milk line.

Calibration and validation of the BROLIS HerdLine analyser were performed at the Eurofins laboratory to ensure its accuracy. The system’s accuracy was evaluated by root mean square error of prediction (RMSEP) values, recorded at 0.21% for fat, 0.19% for protein, and 0.19% for lactose, indicating the dependability of the in-line readings.

### 2.3. Cow Behaviour

At the start of the study, each of the 200 cows was equipped with an orally administered SmaXtec bolus within the first 10 days post-calving. These boluses were inserted into the reticulorumen using a specialised applicator, adhering strictly to the manufacturer’s guidelines. Before deployment, each bolus was activated and linked to the respective cow by cross-referencing its identification number with the ear tag. Additionally, a connection was established with the base station to facilitate continuous data transmission. The SmaXtec boluses continuously monitored key physiological parameters, including reticulorumen pH, temperature, and locomotion activity, allowing real-time data collection. To ensure accuracy, pH calibration was carried out prior to deployment using buffer solutions with pH values of 4 and 7 (Reagecon, Shannon, Ireland). Data were recorded at ten-minute intervals throughout the study period, and all collected information was compiled and displayed using the SmaXtec Messenger^®^ software.

The SmaXtec boluses continuously monitor internal body temperature, reticulorumen pH, activity, and drinking behaviour based on temperature fluctuations after water intake. Data were transmitted wirelessly at 10-min intervals and processed through the SmaXtec Messenger^®^ software. While this system enables non-invasive, real-time collection of physiological data, potential limitations include dependence on regular calibration, possible drift of pH sensors, and the fact that hydration estimates are derived indirectly rather than through direct measurement. The collected data are transmitted wirelessly to a centralised system, allowing farmers and veterinarians to analyse health trends and make informed management decisions. This non-invasive monitoring method enhances herd health management by facilitating early detection of potential health issues and optimising overall productivity.

A wireless implanted device was utilised to measure critical physiological indicators, including reticulorumen temperature, pH, total rumination duration, and physical activity. Data acquisition was facilitated by antennas from SmaXtec Animal Care Technology^®^, while a microprocessor-controlled device recorded pH and TRR readings via an analogue-to-digital (A/D) converter, subsequently archiving the data on an external memory chip for future study. The compiled data were processed and visualised using the SmaXtec Messenger^®^ software (Version 4). This system provided continuous real-time monitoring of health and behavioural indicators. However, while useful for early detection of changes, interpretation of the data requires caution, as sensor accuracy may vary depending on device placement, animal physiology, and farm conditions.

### 2.4. Environmental Parameters

Environmental conditions on the farm were assessed using a SmaXtec climate sensor (SmaXtec Animal Care GmbH, Graz, Austria) installed at cow head height in the centre of the barn to capture the immediate thermal environment experienced by the animals. The sensor continuously recorded ambient temperature (°C) and relative humidity (%) at 10-min intervals. The THI was calculated using the formula:

THI = 0.8 × T + RH × (T − 14.4) + 46.4

where *T* represents temperature (°C) and *RH* denotes relative humidity (%) [35]. A SmaXtec heat stress calculator was utilised to evaluate the effects of heat stress by calculating the heat index. All parameters, including feeding, housing, and milking, remained constant throughout the trial period, with the exception of *THI*. We assessed the effect of heat stress by calculating the average daily *THI* from the continuous data recordings.

To ensure accurate alignment of physiological responses with environmental conditions, each bolus measurement (e.g., reticulorumen pH, temperature) was matched to the concurrent THI reading from the same 10-min interval. This synchronisation allowed for precise evaluation of how changes in THI corresponded with cow physiology in real time. These synchronised datasets were then used to construct the six biologically informed composite threshold conditions (C1–C6).

### 2.5. Data Processing and Condition Definitions

This study employed a multivariate classification framework to assess and predict physiological and metabolic stress states in dairy cows based on sensor-derived internal parameters, environmental conditions, and milk composition traits. The approach combined biological thresholds with advanced ML algorithms to identify risk patterns associated with heat stress, altered rumen function, and shifts in milk quality.

For each condition, cows meeting the defined thresholds were labelled as positive (class 1), while controls (cows exposed to comparable THI but with stable internal responses) were assigned to the negative class (class 0). This design ensured that comparisons were made within equivalent environmental contexts, thus focusing classification on internal physiological responses.

To assess the impact of heat stress and associated metabolic responses in dairy cows, six composite threshold conditions (C1–C6) were formulated based on proven physiological correlations, existing literature, and exploratory investigation of the current dataset. These thresholds were not arbitrarily selected; rather, they were constructed to reflect biologically plausible combinations of environmental exposure and internal physiological or metabolic shifts.

Six biologically meaningful binary classification conditions were defined to represent key states of stress or dysfunction. These included:

Condition 1 (C1) (THI > 73 and milk temperature > 38.6 °C). This condition was established to identify cows experiencing simultaneous external (ambient) and internal (core body) heat stress. A THI value above 73 is widely accepted as indicative of moderate to severe heat stress [25], while milk temperature above 38.6 °C has been associated with increased metabolic activity and thermoregulatory strain [36]. This compound criterion was selected to test whether combined heat load is associated with a shift in reticulorumen pH, potentially reflecting early ruminal acidification.

Condition 2 (C2) (THI > 69 and milk lactose < 4.5%). This condition was developed to investigate whether moderate heat stress is associated with shifts in energy metabolism, as reflected by an elevated milk FPR. A THI > 69 corresponds to mild-to-moderate heat load [37], and a milk lactose concentration < 4.5% has previously been linked to subclinical inflammation or udder health challenges [38]. Although lactose concentration was used to define the heat-affected group, the primary outcome evaluated under this condition was FPR, a known indicator of lipid mobilisation and negative energy balance. The goal was to assess whether cows under moderate thermal stress and potential metabolic disruption exhibit increased FPR, even in the absence of overt clinical symptoms.

Condition 3 (C3) (THI > 70 and rumination time < 420 min/day). This condition was established to evaluate whether behavioural suppression under moderate heat stress is associated with elevated milk temperature, a proxy for internal thermal load. A THI > 70 indicates early to moderate environmental heat stress [39], while rumination time < 420 min/day was chosen based on the lower quartile of the study population and prior evidence linking reduced chewing activity to both heat stress and metabolic imbalance [40]. By using rumination suppression as an entry criterion, this condition allowed investigation into whether milk temperature rises in cows that show early behavioural signs of heat-related discomfort, thus linking external stress, behavioural change, and internal physiological response.

Condition 4 (C4) (THI > 72 and reticulorumen pH < 6.0). This condition was defined to examine whether ruminal acidosis under heat stress is associated with alterations in milk lactose concentration. A reticulorumen pH < 6.0 is widely accepted as indicative of sub-acute ruminal acidosis (SARA) [41], and THI > 72 represents moderate-to-severe environmental heat stress [42]. By evaluating milk lactose levels in cows experiencing both acid–base imbalance and heat stress, this study aimed to determine whether milk lactose could serve as a non-invasive metabolic marker of digestive disruption. Despite the biological plausibility, no significant difference in lactose concentration was observed between groups, suggesting limited sensitivity of this marker under such combined stressors.

Condition 5 (C5) (THI > 70 and milk FPR > 1.5). This condition was constructed to evaluate the relationship between metabolic stress under heat load and milk lactose concentration. Milk FPR above 1.5 is a well-established marker of lipid mobilisation and subclinical ketosis [43,44], while THI > 70 indicates moderate heat stress [37]. The aim was to investigate whether cows experiencing both environmental and metabolic challenges exhibit altered lactose synthesis, potentially due to energy redistribution or underlying inflammation. In this dataset, no significant difference in milk lactose was found, suggesting that lactose may not be sufficiently responsive to reflect early energy imbalance under thermal stress.

Condition 6 (C6) (THI > 74 and milk temperature > 38.7 °C). This condition was defined to identify cows experiencing severe combined heat stress, with the goal of evaluating its effect on rumination time, a sensitive indicator of well-being and feeding behaviour. A THI > 74 reflects high external heat stress [45], while milk temperature > 38.7 °C serves as a marker of internal physiological heat accumulation [46]. Rumination time was used as the outcome variable to determine whether cows exposed to this level of heat stress exhibit behavioural suppression, which may precede clinical signs of thermal distress. The hypothesis was that cows under intense heat exposure would show significantly reduced rumination, supporting its use as a behavioural biomarker for early stress detection.

These composite thresholds were designed not as clinical diagnoses, but as biologically guided categories to evaluate physiological, behavioural, and metabolic responses under realistic farm conditions. Their construction allowed for hypothesis-driven ML classification and statistical testing, bridging environmental exposure with early functional responses in dairy cows.

### 2.6. Feature Selection and Data Preprocessing

The dataset comprised daily records of physiological, behavioural, environmental, and production traits collected from early-lactation dairy cows. Key variables included milk composition (fat, protein, and lactose), rumination time, milk temperature, reticulorumen pH, and external climate data used to calculate the THI. To mitigate model overfitting, feature selection was guided by a combination of variance-based filtering and domain knowledge related to heat stress physiology. Highly collinear predictors were excluded to preserve model stability and interpretability. Multicollinearity among predictors was assessed by calculating pairwise Pearson correlation coefficients. Variables with high correlation (|r| ≥ 0.85) were considered redundant, and the biologically less relevant variable was excluded. In addition, variance inflation factor (VIF) values were inspected, with predictors showing VIF > 10 removed. This procedure ensured that the final set of predictors contributed independent information, improving model stability and interpretability.

All continuous predictors were standardised using a z-score transformation based on the training data to ensure comparability among variables. Missing values were imputed using the median of each variable, derived solely from the training subset, to prevent data leaking during model evaluation. Across the six threshold-based conditions (C1–C6), the number of cows meeting the heat stress criteria was considerably smaller than the control group (C1: 148 vs. 1312; C2: 243 vs. 1217; C3: 126 vs. 1334; C4: 107 vs. 1353; C5: 193 vs. 1267; C6: 157 vs. 1403). To address this imbalance, stratified sampling was applied when splitting data into training (80%) and validation (20%) sets, ensuring proportional representation of positive and control cases. In addition, class weights were incorporated into the loss functions of each algorithm, assigning a higher weight to the minority (heat-stressed) class. This approach prevented bias toward the majority group and ensured that model performance metrics (area under the receiver operating characteristic curve (AUC), Matthews correlation coefficient (MCC), sensitivity, specificity) reflected balanced evaluation. The dataset was randomly partitioned into training (80%) and validation (20%) sets, with stratified sampling applied separately for each binary classification task (C1–C6) to maintain class balance across both sets. This partitioning procedure was repeated ten times using Monte Carlo cross-validation, allowing assessment of model generalisability and stability across different data splits. Model performance metrics were computed for each iteration and then averaged to obtain robust estimates.

Five supervised classification techniques were trained and evaluated: PLS-DA, RF, SVM with a radial basis function (RBF) kernel, NN implemented through a multi-layer perceptron (MLP), and an Ensemble model that integrated predictions from RF, SVM, and NN. Each model was applied independently to all six biologically informed threshold conditions (C1–C6), which represent different physiological and metabolic manifestations of heat stress in dairy cows.

Class weights were specifically calculated by determining the inverse of class frequencies and including them directly into the loss function of each model. This weighting scheme resulted in a greater penalty for misclassifying minority (heat-stressed) cases compared to majority (control) cases, thereby improving sensitivity to biologically relevant stress responses. This approach prioritised the correct classification of minority (positive condition) cases while avoiding information loss associated with under-sampling or artificial distortion of the data through over-sampling techniques. This methodology is consistent with recommendations by Japkowicz and Stephen [47], who emphasise that modifying class weights can achieve effective performance in imbalanced datasets without inflating the sample artificially.

### 2.7. Classification Algorithms

Partial Least Squares Discriminant Analysis (PLS-DA) was applied as a linear, projection-based classification method particularly suitable for high-dimensional and collinear data. This model projects both the predictor variables and the categorical response onto a lower-dimensional latent space, maximising the separation between classes. In this study, PLS-DA was implemented using a one-vs-rest approach, where binary classification was performed for each condition versus its respective control. The optimal number of latent components was determined using cross-validation to balance model complexity and classification performance. PLS-DA provides a straightforward interpretation of variable loadings, offering insight into which physiological or behavioural features contributed most to group separation. Following the methodology described by Barker and Rayens [48], predicted values were thresholded at 0.5, where outputs ≥ 0.5 were classified as condition positive (1) and values < 0.5 as condition negative (0). Model evaluation was conducted using stratified Monte Carlo cross-validation with 10 repetitions to ensure robust performance estimates across varying train-test splits. Standard classification metrics were calculated for each iteration, including AUC, MCC, sensitivity, specificity, positive predictive value (PPV), and negative predictive value (NPV). The number of latent variables (components) in the PLS model was improved by selecting the configuration that maximised AUC during cross-validation. This approach allowed the model to capture relevant multivariate structure in the data while maintaining interpretability and control over model complexity.

Random Forest (RF) [49] was used as a robust ensemble-based learning method that constructs a large number of decision trees during training and outputs the mode of the class predictions. It offers strong resistance to overfitting and can handle non-linear interactions between features without requiring prior transformation. The model was implemented using the Random Forest Classifier from the scikit-learn library. Hyperparameters such as the number of trees (n_estimators), maximum tree depth, and minimum samples per leaf were optimised via grid search with cross-validation. Feature importance was derived from the average decrease in Gini impurity, and model explainability was enhanced using Shapley Additive Explanations (SHAP) to assess the contribution of individual predictors to the final outcome. The Random Forest Classifier was used to develop binary classification models for each of the six threshold-based heat stress conditions (C1–C6), with cows meeting each condition classified against control cows. Each model was executed using 500 trees, utilising the square root of the total number of features chosen at each node division. To rectify class imbalance, class weights were manually calibrated to enhance sensitivity while preserving specificity, assigned as 0.12 for control cows and 0.88 for cows fulfilling each corresponding C-condition. The Gini impurity index was employed to assess feature relevance and determine the most significant predictors for each condition. All RF models, including feature importance estimation, were implemented within the repeated Monte Carlo cross-validation framework to ensure robustness and prevent results from being dependent on a single train–test split.

The Support Vector Machine (SVM) classifier was employed to model non-linear class boundaries through the use of kernel transformations. The radial basis function (RBF) kernel was used due to its flexibility in capturing non-linear patterns within the physiological and behavioural data. The key hyperparameters, including the regularisation parameter C and kernel coefficient gamma, were tuned using grid search with cross-validation. SVM aims to maximise the margin between the classes and is well-suited for medium-sized datasets with complex feature relationships. The decision function was calibrated to provide probability estimates required for AUC and ROC curve computation. Final class weights were set to 0.10 for cows that did not meet the condition (negative class) and 0.90 for cows that did meet the condition (positive class). This weighting strategy prioritised the minority class during model training without introducing synthetic data or reducing sample diversity, thereby enhancing the model’s sensitivity to biologically relevant stress states.

A feed-forward artificial NN [50] was implemented to capture higher-order interactions between predictors through a layered architecture. The model was constructed using the Keras library with a TensorFlow backend. The network consisted of an input layer matching the number of features, one or two hidden layers with ReLU activation, and a final sigmoid-activated output layer for binary classification. Dropout and L2 regularisation were applied to mitigate overfitting. The model was trained using the Adam optimiser with binary cross-entropy loss, with class weights incorporated into the loss function to compensate for the smaller number of heat-stressed cases. Early stopping was applied to prevent overfitting, and performance metrics were averaged across multiple training iterations within the repeated Monte Carlo cross-validation framework to ensure result stability.

To leverage the strengths of multiple classifiers, an ensemble model was constructed using a soft voting strategy. This meta-classifier combined the probabilistic outputs of the RF, SVM, and NN models, weighting them equally. The final prediction was based on the average predicted probability across models, which enhances robustness and generalisation. The ensemble approach was particularly effective in improving predictive accuracy and reducing variance across different conditions (C1–C6). Voting ensemble methods are increasingly used in applied ML due to their ability to stabilise model output in heterogeneous data environments. Specifically, 30% weight was allocated to the Random Forest and 70% to the NN, reflecting their relatively higher predictive accuracy and sensitivity in detecting biologically relevant stress states. This weighting scheme was empirically determined using repeated Monte Carlo cross-validation, where the NN consistently achieved higher sensitivity and AUC compared to the other models. For comparison, equal-weight (simple sum) ensembles were also evaluated but yielded slightly lower performance, particularly in detecting minority (heat-stressed) cases. We acknowledge that the 70/30 weighting strategy is dataset-specific and should be validated in external herds before broader application. This ensemble approach aimed to enhance overall model stability while balancing interpretability, non-linear modelling capacity, and robustness to class imbalance.

To model binary classification for each condition, five commonly used supervised learning algorithms were implemented. PLS-DA was included as a linear model that performs dimensionality reduction by projecting predictor variables into latent structures that maximise covariance with the response variable, followed by logistic regression for final prediction. This method has been widely used in biological and metabolomic studies due to its interpretability and ability to handle multicollinearity.

RF was applied as a tree-based ensemble method utilising bootstrap aggregation and majority voting. Its ability to handle complex non-linear relationships and interactions makes it especially suited for heterogeneous physiological data. A SVM with probability calibration was included for comparison, providing a non-probabilistic model capable of constructing optimal hyperplanes for class separation.

We also implemented a feed-forward NN architecture consisting of a multi-layer perceptron trained using backpropagation with a single hidden layer. Finally, we evaluated a soft-voting Ensemble classifier that combined the predictions of RF, SVM, and NN to leverage the strengths of multiple learners. Although the NN architecture was intentionally simple to avoid overfitting on a moderately sized dataset, it consistently achieved higher sensitivity and AUC during cross-validation compared to the other models. For this reason, the ensemble was constructed with empirically determined weights (RF: 30%, NN: 70%), reflecting validation performance rather than model complexity. This strategy ensured that the ensemble preserved robustness from the RF while enhancing sensitivity through the NN component.

Each model was trained independently for every condition, using the same train-test split and standardised features. Hyperparameters were kept consistent across conditions to preserve comparability, as recommended in best practices for multi-task model evaluation, since tuning each condition separately could introduce unequal model complexity and confound performance comparisons [51]. This ensured that observed performance differences reflected data characteristics rather than hyperparameter choices. Models were trained without internal cross-validation, relying on the separate test set for performance evaluation.

### 2.8. Model Evaluation and Performance Metrics

Model performance was assessed using standard binary classification metrics. Sensitivity (recall), specificity, and overall accuracy were calculated to evaluate the correct detection of positive and negative cases. Positive predictive value (PPV) and negative predictive value (NPV) were computed to reflect the precision of classification. In addition, the area under the receiver operating characteristic curve (AUC) was used to quantify the overall discriminative ability of each model [17]. This modelling approach was designed to support early and biologically meaningful detection of heat stress–related states in dairy cows, offering a scalable and interpretable tool for real-time monitoring and precision livestock management.

To assess the balance between sensitivity and specificity and to account for class imbalance, the MCC was calculated. MCC is considered a robust metric for evaluating binary classifiers, particularly in biologically imbalanced settings, as it incorporates all elements of the confusion matrix [17]. Chicco and Jurman [52] assert that the MCC is an alternative statistic unaffected by imbalanced data. The author characterised it as the calculus of the Pearson product-moment correlation coefficient between the observed and expected values. The measure’s range is set to [−1, +1]. When the MCC is near 1, the model is capable of producing predictions that are exceedingly precise. However, the model’s efficacy is substandard when the MCC is near −1. An MCC of zero suggests that the prediction is no more precise than a random one [53]. The MCC was selected as a critical metric, following Chicco and Jurman [52], due to its ability to provide a comprehensive assessment of classification performance and its reduced sensitivity to class imbalance. The classification threshold was set at 0.5, and AUC values were calculated from ROC curves using predicted probabilities.

To provide stable estimates and assess the reliability of each model, all metrics were calculated across 100 bootstrap iterations using the test set. For each model and condition, the mean and standard deviation (mean ± SD) of all metrics were reported [17].

### 2.9. Implementation and Software

A supervised classification framework was constructed in Python (version 3.10) [54] to assess the efficacy of ML models for the early diagnosis of subclinical heat stress in dairy cows. The libraries employed are: scikit-learn (version 1.4.0) [55], pandas (version 2.2.1) [56], matplotlib (version 3.8.0) [57], SciPy (version 1.12.0) [58], and Keras [59] utilising TensorFlow backend (version 2.15.0) [60]. All calculations were performed on a high-performance local computer system. The modelling pipeline employed widely recognised scientific computing tools, such as scikit-learn for traditional machine learning algorithms and assessment measures, Keras with a TensorFlow backend for neural network implementation, and matplotlib and seaborn for data visualisation [61]. All procedures followed established best practices for supervised classification, including data standardisation, stratified train–test splitting, handling of class imbalance with class weights, prevention of data leakage during imputation, repeated Monte Carlo cross-validation, and evaluation using multiple performance metrics (AUC, MCC, sensitivity, specificity, PPV, NPV), with a specific focus on a multi-condition binary framework based on six biologically defined threshold conditions (C1–C6). Each condition represented a unique physiological or metabolic stress profile, treated independently as a binary classification task (1 = condition met; 0 = condition not met). This structure allowed the models to evaluate the likelihood of specific subclinical states arising from heat stress.

Table 3 provides an overview of the structural characteristics of the ML models applied in this study to classify cows according to six biologically defined threshold conditions (C1–C6). Each model is described in terms of its algorithmic type, key hyperparameters, interpretability approach, and architectural design. The linear model (PLS-DA) was included to support projection-based classification using latent variables. Tree-based models (RF) and kernel-based approaches (SVM) offered complementary perspectives on non-linearity and decision boundaries. A feedforward NN architecture with two hidden layers was employed, optimised using the Adam algorithm and configured with early stopping and dropout to prevent overfitting. An ensemble model combining RF and NN outputs via weighted voting (RF: 30%, NN: 70%) was also tested to improve robustness and generalisation. Feature importance and interpretability were assessed using permutation-based methods and SHAP, where applicable. Training and evaluation were conducted on standardised physiological and milk data collected from dairy cows during the first 60 days postpartum, allowing for consistent threshold classification across all model types.

### 2.10. Statistical Analysis

Statistical analyses were conducted using IBM SPSS Statistics 29.0 (IBM Corp., Armonk, NY, USA) to evaluate the impact of heat stress on physiological, behavioural, and production parameters in dairy cows. Data points were grouped according to the average daily THI at the time of each recording. For bolus-derived variables, 10-min interval measurements were matched with corresponding THI values from the same time window. These matched datasets were then averaged by THI group per cow to enable comparative analysis across different thermal load categories. Data normality was assessed using standard diagnostic procedures. For variables that followed a normal distribution, group comparisons across the six threshold-based conditions (C1–C6) were performed using one-way analysis of variance (ANOVA). To explore linear relationships between environmental, physiological, and production parameters, Pearson’s correlation analysis was applied. Correlation strength was interpreted as follows: weak (|r| < 0.3), moderate (|r| = 0.3–0.7), and strong (|r| > 0.7). Correlations were considered statistically significant at *p* < 0.05 and highly significant at *p* < 0.01.

## 3. Results

The performance metrics of PLS-DA, RF, SVM, NN, and an Ensemble model are summarised in Table 4. Among all models, PLS-DA demonstrated the highest overall performance, with an accuracy of 66.7%, sensitivity of 0.50, specificity of 0.81, and area under the ROC curve (AUC) of 0.65. It also achieved the highest MCC of 0.33, indicating good agreement between predicted and actual classifications. In contrast to PLS-DA, the ensemble and neural network models performed less reliably, showing lower sensitivity, specificity, and overall accuracy. This reduced performance is likely attributable to the limited number of low-pH cases and resulting class imbalance, which disproportionately affected models requiring larger balanced datasets. The Random Forest model provided only moderate predictive value (accuracy = 53.3%), while the SVM demonstrated marginal discrimination ability (AUC = 0.54). These results highlight the challenge of reliably detecting early ruminal acidosis under C1 when sample sizes are small and class imbalance is pronounced.

To evaluate the predictive value of milk quality and physiological traits under subclinical heat stress, we trained five classification models to predict an elevated milk FPR (milk FPR > 1.5), which may reflect inflammatory or metabolic stress. The models were applied to a filtered dataset (Condition C2: THI > 69 and milk lactose < 4.5%), using features such as THI, lactose and milk FPR (Table 5).

The NN model showed the best sensitivity (0.30 ± 0.14) and MCC (0.33 ± 0.18), suggesting some potential to detect elevated FPR in imbalanced data. However, most models, including Random Forest and SVM, classified almost all cows as non-stressed (low FPR), yielding high specificity but zero sensitivity. The PLS-DA model showed perfect specificity and high PPV (1.00), but with limited ability to detect positive cases (sensitivity = 0.10).

To predict cows at risk of elevated milk temperature under behavioural suppression (Condition C3: THI > 70 and Rumination < 420 min/day), five supervised ML classifiers were developed using integrated physiological, behavioural, and milk composition features. As shown in Table 6, all models demonstrated robust classification performance.

The Ensemble model achieved the highest overall metrics (Accuracy: 0.90 ± 0.01; AUC: 0.95 ± 0.01; MCC: 0.81 ± 0.02), closely followed by the Random Forest model (Accuracy: 0.87 ± 0.01; AUC: 0.93 ± 0.02). Neural Network and SVM models showed balanced sensitivity and specificity, though with slightly lower predictive strength. PLS-DA had the lowest performance among the models but still offered meaningful classification above chance levels (Accuracy: 0.71 ± 0.02).

To predict cows at risk of elevated milk lactose under heat stress (Condition C4: THI > 72 and reticulorumen pH < 6.0), five supervised ML classifiers were developed using integrated physiological and milk quality features (Table 7).

The Ensemble model showed the best overall performance (Accuracy: 0.96 ± 0.03; MCC: 0.92 ± 0.05; AUC: 0.99 ± 0.01), closely followed by PLS-DA and Neural Network, which also provided strong classification results with balanced sensitivity and specificity.

Although Random Forest reached perfect scores across all metrics, such performance may reflect overfitting or high feature separability in the training set and should be interpreted with caution unless externally validated.

In this analysis, to predict cows at risk of elevated milk lactose under heat stress (Condition C5: THI > 70 and milk FPR > 1.5), we assessed the capacity of ML models to detect cows with elevated milk FPR (milk FPR > 1.5) during periods of high heat load (THI > 70) (Table 8).

The RF model achieved the highest performance across all metrics, with perfect specificity (1.00), sensitivity (0.97), and AUC (1.00), suggesting strong discriminative ability for this biomarker threshold. The NN, PLS-DA, and Ensemble models also performed exceptionally well, with high accuracy (≥0.97) and MCC values above 0.93.

Although SVM achieved perfect specificity and precision, its sensitivity (0.82) was slightly lower, indicating potential under-detection of positive cases. Overall, the results suggest that FPR prediction under heat stress is highly feasible using physiological, behavioural, and milk composition features.

Condition C6, which focuses on identifying cows under combined internal and external heat stress (THI > 74 and milk temperature > 38.7 °C) to predict cows at risk of lower rumination time under heat stress (Table 9).

To evaluate the risk of combined heat stress, we analysed cows exposed to both elevated environmental heat (THI > 74) and increased milk temperature (>38.7 °C). Five ML models were trained.

The RF, NN, and Ensemble models all achieved perfect performance across all metrics (accuracy, sensitivity, specificity, AUC = 1.00), suggesting a clear signal in the data that separates high heat stress cases from controls. However, these results should be interpreted cautiously and validated externally to rule out overfitting.

The PLS-DA model also performed strongly, with high specificity (1.00), sensitivity (0.83 ± 0.11), and MCC (0.77 ± 0.12), while SVM underperformed relative to the others, with lower specificity (0.60 ± 0.24) and MCC (0.43 ± 0.25).

Descriptive statistics were calculated for six predefined threshold-based conditions (C1–C6), each targeting a different milk or physiological parameter affected by heat stress or metabolic imbalance in dairy cows. The comparison was made between two groups: cows that met the threshold condition (Condition Met) and those that did not (Control). The table presents means, standard deviations (SD), standard errors of the mean (SEM), 95% confidence intervals (CI), minimum and maximum values, Cohen’s d effect sizes, and *p*-values from independent *t*-tests (Table 10).

Under Condition C1 (THI > 73 and milk temperature > 38.6 °C), cows exhibited a lower reticulorumen pH (mean = 6.03, SD = 0.28) compared to controls (mean = 6.09, SD = 0.29), with a statistically significant difference (*p* = 0.013) and a small effect size (Cohen’s d = –0.212).

In Condition C2 (THI > 69 and milk lactose < 4.5%), cows showed a significantly higher milk FPR (mean = 1.28, SD = 0.29) than controls (mean = 1.20, SD = 0.31), with a *p*-value of 0.0002 and a small-to-moderate effect size (Cohen’s d = 0.258).

Condition C3 (THI > 70 and rumination < 420 min/day) was associated with elevated milk temperature in the affected group (mean = 38.77 °C, SD = 0.42) versus the control group (mean = 38.52 °C, SD = 0.34). This difference was highly significant (*p* < 0.00001) with a large effect size (Cohen’s d = 0.637), indicating that behavioural suppression under stress may precede systemic overheating.

In contrast, Condition C4 (THI > 72 and reticulorumen pH < 6.0) did not show a significant difference in milk lactose concentration between the Condition Met group (mean = 4.58%, SD = 0.39) and the control group (mean = 4.63%, SD = 0.42), with a *p*-value of 0.243 and a negligible effect size (Cohen’s d = −0.114). Similarly, Condition C5 (THI > 70 and milk FPR > 1.5) failed to show a significant reduction in milk lactose (*p* = 0.443; Cohen’s d = −0.062), suggesting that lactose concentration was not a sensitive marker for inflammation or acidosis in this dataset.

Finally, in Condition C6 (THI > 74 and milk temperature > 38.7 °C), cows had substantially lower rumination times (mean = 424.41 min, SD = 85.03) compared to controls (mean = 453.56 min, SD = 84.39). The difference was statistically significant (*p* = 0.013) with a moderate effect size (Cohen’s d = −0.344), supporting the hypothesis that high heat load impairs feeding and rumination behaviour.

**Figure 1:**
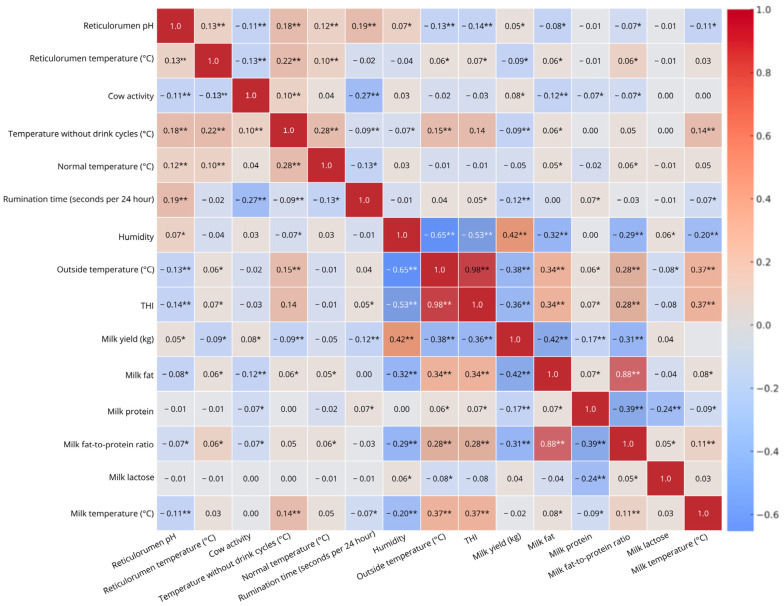
Correlation heatmap of dairy cow indicators with statistical significance markers. ** Correlation r (Pearson’s correlation coefficient) is significant at *p* < 0.01; * Correlation is significant *p* < 0.05.

The correlation heatmap highlights significant relationships between various indicators. A weak positive correlation (r = 0.22) between reticulorumen temperature and temperature without drink cycles (*p* < 0.001) suggests a slight but meaningful link. Similarly, temperature without drink cycles and normal temperature show a weak positive correlation (r = 0.28, *p* < 0.001), indicating that these temperature measures are related. A weak negative correlation (r = −0.27, *p* < 0.001) between cow activity and rumination time suggests that increased activity is associated with reduced rumination. Strong negative correlations were observed between humidity and outside temperature (r = −0.65, *p* < 0.001) and between humidity and THI (r = −0.53, *p* < 0.001), confirming that increased temperature lowers humidity and affects the thermal heat index. Interestingly, humidity was moderately positively correlated with milk yield (r = 0.42, *p* < 0.001) but negatively correlated with milk fat (r = −0.32, *p* < 0.001) and milk FPR (r = −0.29, *p* < 0.001), suggesting that humid conditions may promote milk production while slightly reducing fat content. A strong positive correlation (r = 0.98, *p* < 0.001) between outside temperature and THI confirms that THI is highly temperature-dependent, while a moderate negative correlation (r = −0.38, *p* < 0.001) between outside temperature and milk yield suggests that hotter conditions may reduce milk production. However, outside temperature was moderately positively correlated with milk fat (r = 0.34, *p* < 0.001) and milk temperature (r = 0.37, *p* < 0.001), and weakly correlated with the milk FPR (r = 0.28, *p* < 0.001), indicating a potential effect of heat stress on milk composition. Similarly, THI showed a moderate negative correlation with milk yield (r = −0.36, *p* < 0.001) but a moderate positive correlation with milk fat (r = 0.34, *p* < 0.001), the milk FPR (r = 0.28, *p* < 0.001), and milk temperature (r = 0.37, *p* < 0.001), reinforcing the impact of heat stress on dairy production. A moderate negative correlation between milk yield and milk fat (r = −0.42, *p* < 0.001) and between milk yield and the FPR (r = −0.31, *p* < 0.001) suggests that higher milk production is associated with lower fat concentration. In contrast, a strong positive correlation (r = 0.88, *p* < 0.001) between milk fat and the FPR highlights the strong relationship between these milk composition measures. Additionally, a moderate negative correlation between milk protein and the FPR (r = −0.39, *p* < 0.001) and a weak negative correlation between milk protein and milk lactose (r = −0.24, *p* < 0.001) suggest inverse associations between these milk quality indicators (Figure 1). 

## 4. Discussion

### 4.1. Physiological Significance and Machine Learning Insights from Threshold-Based Heat Stress Detection

Data obtained from precision livestock farming (PLF) devices can facilitate the modelling of heat stress effects, including its influence on rumen function, and support the implementation of suitable mitigation strategies [62]. This study introduces a novel integrative framework combining biologically defined threshold conditions (C1–C6) with supervised ML to detect early physiological and behavioural alterations associated with heat stress in dairy cows. The results confirm that heat stress has measurable effects on reticulorumen pH, rumination time, milk temperature, and metabolic indicators such as milk FPR and lactose. This study evaluated the capacity of five supervised ML algorithms—PLS-DA, RF, SVM, NN, and an Ensemble model—to classify physiological and metabolic disturbances in dairy cows under various heat stress conditions. Using a combination of environmental (THI), physiological (milk temperature, rumination), and milk composition parameters (milk lactose, FPR), we explored how different models perform in detecting ruminal acidosis, elevated milk FPR, and suppressed rumination, which are indicative of systemic stress and reduced welfare. Our results demonstrate that model performance varied depending on the targeted physiological condition, the features included, and the degree of class balance. Among the algorithms tested, PLS-DA, RF, and the Ensemble models consistently achieved higher performance across multiple scenarios compared to SVM and NN. Becker et al. [12] created models for the classification of stress in dairy cows utilising three distinct algorithms (random forest, logistic regression, and Gaussian Naive Bayes) and four types of thermal comfort interventions, achieving accuracies ranging from 81.1% to 89.3% [12]. Rodrigues et al. [63] developed a unique infrared thermography feature extraction approach for dairy cow heat stress classification [63]. The thermal signature approach uses the frequency of each predefined temperature range in the thermographic images’ temperature matrix to build an IRT data descriptor vector. Images were taken from five animal body areas. The thermal signature and environmental data were used to build ANN models. The optimal model, derived from the thermal signature of the ocular region, achieved an accuracy of 90.1%, surpassing the accuracy of the current study’s most effective model (86.8%) [63]. Given Rodrigues et al. [63] higher accuracy on the same dataset, the thermal signature method may have been the main reason for model improvement [63].

Under C1 (THI > 73 and milk temperature > 38.6 °C), the physiological hypothesis was supported by a statistically significant reduction in reticulorumen pH (*p* = 0.013), albeit with a small effect size (Cohen’s d = −0.21). Among ML models, PLS-DA achieved the highest classification performance (accuracy = 66.7%, AUC = 0.65), while RF and SVM performed modestly. This suggests that linear models like PLS-DA may better capture subtle shifts in physiological states under moderate heat stress when feature variance is low. However, lower sensitivity across all models (≤0.50) reflects the difficulty of detecting early-stage acidosis, particularly in the presence of class imbalance and limited variance in input features. Wagner et al. [64] submitted 14 cows (Bos taurus) to SARA, a condition that might cause behavioural alterations. A further 14 control cows were not submitted to SARA. K Nearest Neighbours for Regression (KNNR), Decision Tree for Regression (DTR), Multi-Layer Perceptron (MLP), Long Short-Term Memory (LSTM), and an algorithm that assumes daily activity is similar were tested. The best SARA detection algorithm, KNNR, detected 83% of true-positives but created 66% of false-positives, limiting its practical usage. Applying ML to huge animal datasets rather than group datasets may yield more gains [64]. T. Touil et al. [65] showed in their work that SARA can be accurately predicted from milk Fourier-transform infrared (FTIR) spectroscopy data utilising ML models. Through the examination of milk samples from individual cows on 12 commercial farms and the use of several ML algorithms—specifically, random forest, gradient boosting, and partial least squares—the researchers attained prediction accuracies for SARA of up to 69% in external validation contexts [65]. Real-time monitoring of pH and temperature in the reticular contents of dairy cows has been proposed as an effective tool for evaluating the risk of subclinical acidosis [66]. The pH of the reticulorumen is more likely to be lower in cows who are more susceptible to heat stress [67]. SARA typically arises when ruminal pH remains between 5.2 and 6 for an extended duration [68,69]. Numerous research studies have emphasised the correlation between heat stress vulnerability and reduced reticulorumen pH. For example, research undertaken by Shengguo Zhao and colleagues [70] demonstrated that the concentration of rumen acetate and pH in the heat-stressed group was substantially reduced, while the concentration of ruminal lactate increased. Cows experiencing heat stress tend to reduce their feed intake, which in turn leads to less rumination. This decrease in rumination lowers the production of saliva, a key source of buffering agents for the rumen [71]. Moreover, heat stress causes blood flow to be redirected toward the extremities to enhance heat loss, which reduces the blood supply to the gastrointestinal tract. As a result, the absorption of digestion by-products, such as volatile fatty acids (VFAs), becomes less efficient, leading to a rise in total rumen VFA concentration and a corresponding decrease in pH. Additionally, the increased respiratory rate during heat stress contributes to rumen acidosis. Panting results in greater CO_2_ exhalation, and since the body relies on a specific bicarbonate (HCO_3_) to CO_2_ ratio for blood pH buffering, the reduction in blood CO_2_ levels prompts the kidneys to excrete bicarbonate. This response limits the amount of HCO_3_ available for buffering in the rumen, exacerbating the drop in pH. Furthermore, panting cows tend to drool more, which reduces the saliva available for the rumen. The combination of reduced bicarbonate content in saliva and the diminished saliva entering the rumen makes cows under heat stress more vulnerable to both subclinical and acute rumen acidosis [72]. Thus, maintaining a healthy rumen is essential for any type of nutritional intervention to counteract the effects of heat stress [72]. Farmers can assist their cows in better managing the challenges of heat stress and preventing potentially severe health issues by ensuring a healthy reticulorumen environment, whether through dietary adjustments or other interventions. It is imperative to effectively manage heat stress in order to optimise the efficacy of production and the welfare of cows.

Milk FPR is a relevant marker for energy balance and subclinical inflammation. Under C2 (THI > 69, milk lactose < 4.5%), cows had significantly higher milk FPR values (*p* < 0.001, d = 0.26), but ML models struggled to identify cows with high FPR due to poor sensitivity (e.g., SVM and RF = 0.00). Notably, only the NN model reached an MCC > 0.30, likely due to its ability to model non-linear interactions between heat load and milk biosynthesis. According to Alicja Satoła et al. [73], using the fat-to-protein ratio as the sole variable in models for predicting subclinical ketosis proved inadequate, as the models achieved only moderate sensitivity (ranging from 0.58 to 0.69) and specificity (ranging from 0.66 to 0.71) [73]. Heat stress modifies metabolites in the mammary glands of lactating dairy cows, impacting glycolysis, lactose, ketone, tricarboxylic acid cycle, amino acid, and nucleotide metabolism, thereby impeding the availability of essential components for milk production in lactating Holstein cows [74]. Oxidative stress is exacerbated by heat stress, which alters the metabolic and molecular activity of mammary secretory tissue. This results in a decrease in cellular efficacy for the synthesis of milk components and a change in the composition of milk [1]. In our study, outside temperature was moderately positively correlated with milk fat (r = 0.34, *p* < 0.001) and milk temperature (r = 0.37, *p* < 0.001), and weakly correlated with the milk FPR ratio (r = 0.28, *p* < 0.001), indicating a potential effect of heat stress on milk composition. Jang-Hoon Jo et al. [75] reported analogous research findings, indicating that milk fat is elevated throughout summer, maybe due to prolonged heat stress, resulting in the breakdown of long-chain fatty acids and consequently, a rise in milk fat [75]. Moreover, the elevated fat percentage in the milk of heat-stressed cows may be ascribed to the decreased milk supply and resultant fat concentration, together with potentially increased non-protein nitrogen levels in the milk from cows experiencing heat stress [75]. The alterations in milk composition resulting from heat stress may affect dairy farmers regarding milk quality and production. Farmers must monitor and control heat stress in their cows to preserve optimal milk composition and overall herd health. Additional research may be required to comprehensively elucidate the processes behind these alterations and to design ways to alleviate the impact of heat stress on milk production.

In contrast, when the milk FPR threshold was adjusted to >1.5 in C5, all models achieved high predictive performance. The RF, Ensemble, and NN models yielded AUCs ≥ 0.999 and MCC values > 0.93, highlighting the importance of optimising threshold selection in biomarker-based models. However, no substantial difference in milk lactose concentration was observed between the C2 and C5 groups (*p* > 0.24), indicating that lactose is not a sensitive stand-alone marker of stress-related metabolic imbalance, at least in the early phases. These results suggest that, although individual biomarkers such as lactose may not be sufficient to identify stress-related metabolic disturbances on their own, their accuracy can be significantly improved by incorporating them into a predictive model in conjunction with other markers. This emphasises the significance of employing a multi-marker approach to create effective diagnostic instruments for the early detection of metabolic disorders in dairy cows. Further research is needed to identify additional biomarkers that can complement lactose in improving the predictive performance of these models.

In C3 (THI > 70, rumination < 420 min/day), affected cows exhibited significantly higher milk temperatures (*p* < 0.00001, d = 0.64). This finding confirms the link between behavioural suppression and thermoregulatory strain, where reduced feed intake leads to lower buffering capacity and greater internal heat retention. ML models, especially the Ensemble and RF classifiers, achieved high accuracy (≥0.87) and AUC values (>0.93), demonstrating their potential to detect behavioural manifestations of heat stress with high reliability. Dairy cows respond to heat stress through physiological adaptations, including peripheral vasodilation, which increases blood flow to the skin, raising skin temperature and enhancing heat dissipation. When body temperature rises due to inadequate heat dissipation, cows compensate by increasing perspiration to improve cooling efficiency. To maintain a stable body temperature, dairy cows regulate blood circulation and facilitate thermal exchange between their core and epidermis [76]. Gonzalez-Rivas et al. [77] report a strong positive association between rumen temperature and THI. Consistent with our research, Liang et al. [78] noted that climate conditions had a more pronounced effect on rumen temperature during summer (40.4 °C) compared to spring and autumn (40.1 °C) or winter (40.0 °C). The heat produced during fermentation causes the temperature in the rumen of dairy cows to be typically elevated compared to other body regions [75]. The temperature in the rumen continues to rise due to heat stress, and there are changes in the feed intake and the microbial compositions in the body to reduce heat generation [75].According to J.W. West et al.’s [79] findings, milk temperature rose steadily with the outside temperature, reaching 39 °C when the THI hit 72, the point at which cows’ feed intake and milk production decreased, and other signs of heat stress manifested. This suggests that milk temperature can be a useful tool for evaluating heat stress [25,79]. Monitoring milk temperature can help farmers identify and address heat stress in their herd before it leads to more serious health issues. By using milk temperature as an indicator of heat stress, producers can implement strategies to mitigate its effects and ensure the well-being of their cows.

Despite no significant group difference in milk lactose concentration (*p* = 0.24), classification performance under C4 (THI > 72 and reticulorumen pH < 6.0) was excellent. RF, NN, and Ensemble models reached perfect or near-perfect performance across all metrics (AUC = 0.99–1.00; MCC > 0.92). This highlights that multi-modal integration of THI, milk temperature, and composition can overcome the limitations of single-variable markers. However, the exceptionally high classification scores may also reflect overfitting, which must be addressed with external validation in larger, independent datasets. Heat stress not only diminishes milk quantity but also negatively influences milk quality, resulting in reduced amounts of lactose [2]. H. M. Farrell et al. [80] noticed that the lactose levels in grazing cattle diminish throughout the summer season in comparison to the winter season. Garner et al. [81] have demonstrated that cows that are subjected to prolonged heat exposure generate milk with a composition that is up to 49% lower in lactose. During heat stress, the lactose content in dairy cow milk decreases due to disruptions in lactose synthesis within the mammary gland. Heat stress induces physiological changes, including elevated cortisol levels and oxidative stress, which can downregulate α-lactalbumin (α-La) synthesis—a crucial coenzyme for lactose production. Since α-La modifies the substrate specificity of β-1,4-galactosyltransferase, its reduction impairs the formation of the lactose synthase enzyme complex in the Golgi apparatus of mammary epithelial cells, leading to decreased lactose synthesis [2,82]. Additionally, heat stress alters glucose metabolism by increasing energy demands for thermoregulation, potentially reducing glucose availability for lactose formation. Collectively, these factors contribute to lower lactose concentrations in milk under heat stress conditions.

C6 combined environmental (THI > 74) and internal (milk temperature > 38.7 °C) heat stress thresholds to predict lower rumination. Affected cows showed significantly lower rumination times (*p* = 0.014, d = −0.34), indicating compromised feeding behaviour under acute heat stress. ML models—particularly RF, NN, and Ensemble—achieved perfect classification accuracy, again raising the need for cautious interpretation. Nonetheless, the strong signal separation affirms the feasibility of developing real-time monitoring tools to detect compound stressors, especially when leveraging integrated biosensor platforms. Ungar et al. [83] utilised discriminant analysis, logistic regression, and NN as classification techniques, reporting accuracy rates of 67% to 82%, 87%, and 25% to 90%, respectively, in the accurate classification of jaw statistics [83]. Giovanetti et al. [84] effectively employed stepwise discriminant analysis (SDA), canonical discriminant analysis (CDA), and discriminant analysis (DA) to autonomously assess particular behaviours utilising a triaxial accelerometer, including the biting activity of dairy sheep in grazing settings [84]. Abdanan Mehdizadeh et al. [85] reported that the accuracy of predicting grazing, ruminating, and resting behaviours ranged from 89% to 95%, yielding an overall accuracy of 93% [85]. Chelotti et al. [86] developed a pattern recognition technique to classify jaw movements in grazing cattle using acoustic data, achieving a detection rate of 90% in noisy environments [86]. Ayadi et al. [87] present a novel monitoring technique utilising a convolutional neural network (CNN)-based deep learning models in their investigation. The classification technique is executed under two primary categories: ruminating and others, utilising all bovine postures recorded by the monitoring camera. The approach proposed by Ayadi et al. [87] is straightforward and user-friendly, capable of capturing long-term dynamics through a condensed representation of a video in a single 2D image. This method demonstrated efficacy in identifying rumination behaviour, with average accuracy, recall, and precision rates of 95%, 98%, and 98%, respectively [87]. Moreover, research conducted by Antanaitis et al. [33] revealed that heat stress substantially reduced ruminating time by up to 70% in cows categorised within the highest THI range (73 to 78) and increased body temperature by 2%. It resulted in a 12.6% decrease in partial carbon dioxide pressure (pCO_2_) and a 32% increase in partial oxygen pressure (pO_2_). Additionally, plasma sodium and potassium decreased by 1.36% and 6%, respectively, while chloride increased by 3% [33]. Recent research has found the effects of heat stress on reticulorumen parameters, increasing the risk of acidosis and influencing the activity levels of cows. Heat stress negatively impacted reticulorumen pH, temperature, and the rumination index. A heightened THI (≥72) increases the risk of ruminal acidosis and influences the physical activity levels of cows [88]. The results demonstrate that Smaxtec data can be employed to assess and analyse the impact of heat stress on dairy cows. These real-time data enable the prompt detection of heat stress effects, reducing the impact of measurement uncertainty and stress associated with animal handling.

The RF and Ensemble models consistently achieved high accuracy and AUC in classifying heifers into threshold-defined states, demonstrating the effectiveness of ML models. It is crucial to note that the models were not black boxes, but rather biologically traceable, as disclosed by SHAP-based interpretability. This highlights the importance of features such as milk temperature, rumination, and pH as significant contributors. This implies a practical relevance for real-time farm monitoring systems that are designed to anticipate at-risk animals before clinical signs appear.

Taken together, these findings support the development of sensor-integrated, threshold-based monitoring systems that move beyond static indicators toward personalised animal-level diagnostics. Further studies should validate the generalisability of the C1–C6 framework across seasons, housing systems, and breeds, and assess its effectiveness in real-time interventions on commercial farms. Prior research has validated the accuracy and reliability of intra-ruminal sensors, further supporting their use in PLF for heat stress management [89]. By utilising continuous physiological monitoring, dairy farmers can implement data-driven decision-making, optimising cow welfare and milk production efficiency.

Beyond individual herd-level applications, the proposed threshold-based classification framework also has potential implications for bioclimatic zoning and climate adaptation strategies. By integrating environmental variables (THI, ambient temperature, humidity) with internal physiological and behavioural indicators, the methodology can be extended to spatial modelling systems that predict regional heat stress risk. Such models could be incorporated into bioclimatic zoning tools to identify high-risk areas for dairy production under current and projected climate conditions. This approach aligns with recent studies emphasising the need to combine multiple data streams for spatially explicit risk assessment and management [90,91]. In this context, our framework not only contributes to on-farm early warning systems but also offers a scalable methodology to support climate-resilient livestock planning, enabling producers and policymakers to anticipate shifts in thermal load patterns and adapt housing, nutrition, and management strategies accordingly.

### 4.2. Study Strengths, Methodological Limitations, and Directions for Future Research

While this study advances the integration of biologically informed threshold conditions with ML for early detection of heat- and metabolism-related stress in dairy cows, several limitations warrant consideration. First, all data were sourced from a single commercial dairy farm with Holstein-Friesian cows managed under specific housing and feeding systems during the early postpartum period (0–60 days in milk). As such, the generalisability of our threshold conditions (C1–C6) and model performance to other breeds, management systems, climatic regions, or lactation stages remains to be evaluated. Replication across multiple farms and seasons is necessary before implementing these thresholds in broader precision farming platforms.

Second, although the thresholds were grounded in established physiological knowledge and exploratory data analysis, they have not yet been validated against gold-standard measures of animal health, such as blood biomarkers, rumen fluid sampling, or clinical diagnoses. For example, while reticulorumen pH below 6.0 is a recognised indicator of sub-acute ruminal acidosis in literature, our study relied on sensor-derived estimates without corroborating laboratory validation. Similarly, deviations in milk lactose and FPR may reflect inflammation or metabolic imbalance, but are influenced by myriad factors, including stage of lactation and dietary changes.

Third, some of the algorithmic results—particularly the near-perfect accuracy and AUC values achieved by RF and Ensemble models—suggest potential overfitting. Although we performed stratified train–test splits and bootstrap resampling, external validation on independent datasets is essential to confirm model robustness and avoid results artificially inflated by data idiosyncrasies.

Fourth, while explaining model decisions using SHAP values enhances interpretability, the resulting feature contributions may be overly specific to our dataset. Breeds, feed formulations, sensor models, or farm management practices not represented here could alter these associations. Future work should evaluate whether the same features hold predictive weight in other contexts or require adaptation.

Finally, although our dataset was rich—with 200 cows monitored for 60 days and approximately 36,000 records—the non-random missing data or sensor failures could introduce biases in both model training and results interpretation. While we employed standard preprocessing methods to handle missing values and outliers consistently, future studies may consider alternative imputation strategies or sensitivity analyses to quantify the impact of missingness on threshold detection and model performance. Missing values were imputed using the median of each variable, calculated from the training set only to avoid data leakage, while outliers were handled through winsorisation at the 1st and 99th percentiles [92]. These preprocessing steps minimised the influence of missing or extreme values while preserving the integrity of the dataset.

In conclusion, while the proposed thresholds and models show great promise for early detection of heat and metabolic stress in dairy herds, further research is required to validate these tools across diverse production environments, health statuses, and lactation stages to ensure their robustness and practical applicability in real-world decision-support systems.

## 5. Conclusions

This study demonstrated that biologically constructed threshold conditions (C1–C6), integrating environmental, physiological, behavioural, and milk composition parameters, are effective for identifying early signs of heat stress and metabolic imbalance in dairy cows during the early postpartum period. Statistically significant differences in reticulorumen pH, milk FPR, milk temperature, and rumination time were observed in cows meeting selected threshold conditions, supporting their biological relevance.

The application of five supervised ML models further validated the predictive power of these conditions. The Random Forest and Ensemble models showed the highest classification performance across multiple metrics, while SHAP-based interpretability provided transparent insights into feature contributions. This integrated approach not only enhances the early detection of subclinical stress responses but also provides a reproducible framework that connects biological comprehension with data-driven technologies.

The results emphasise the potential of threshold-based classification in conjunction with ML for applying to precision livestock husbandry. Future work should focus on validating these models in independent herds, extending monitoring across lactation stages, and integrating real-time sensor data for on-farm decision support systems.

## Figures and Tables

**Table 1:** TMR diet composition for cows.

TMR Component	Value (%)
Corn silage	24
Grass hay	5
Grass silage	16
Grain concentrates slurry	50%
Mineral supplements	5%

**Table 2:** Nutrient composition of the TMR diet.

TMR Component	Value (%)
Dry matter content	48
Acid detergent fibre	20
Neutral detergent fibre	28
Non-fibre carbohydrates	39
Crude protein	16

**Table 3:** Summary of machine learning models, key parameters, and interpretability strategies applied for threshold-based health classification in dairy cows.

Model	Type	Key Parameters	Interpretability (e.g., SHAP)	Architecture Summary
PLS-DA	Linear projection	n_components = 2	Feature loadings per component (PLS)	Latent variable-based classification
RF	Tree-based	n_estimators = 500 trees, max_features = square root of total predictors (√p), class weight manually adjusted (0.12 control, 0.88 heat-stressed)	Gini-based feature importance, SHAP used	Bagged decision trees for binary classification
SVM	Non-linear kernel	Kernel = ‘rbf’, C = 1.0, gamma = 0.1, class weight = balanced	Permutation importance, SHAP used	Margin-based classification using RBF kernel
NN	Deep learning	2 hidden layers (32, 2 neurons), activation = [ReLU, sigmoid], optimiser = Adam	Permutation importance, SHAP used	Multi-layer perceptron (MLP), early stopping, dropout
Ensemble	Hybrid aggregated	Weighted voting: RF (30%) + NN (70%)	Aggregated SHAP from RF, SVM, NN	Integrated predictions from RF, SVM, and NN

MLP—multi-layer perceptron, SHAP—Shapley additive explanations, PLS-DA—partial least squares discriminant analysis, RF—random forest, SVM—support vector machine, NN—neural network, Ensemble—integrated model combining predictions from RF, SVM, and NN.

**Table 4:** Performance metrics of five classification models for detecting low reticulorumen pH (<6.0) under heat stress condition (C1).

Model	Sensitivity	Specificity	Accuracy	PPV	NPV	AUC	MCC
PLS-DA	0.50 ± 0.13	0.81 ± 0.09	0.67 ± 0.08	0.70 ± 0.12	0.65 ± 0.07	0.65 ± 0.10	0.33 ± 0.16
RF	0.50 ± 0.13	0.56 ± 0.11	0.53 ± 0.08	0.50 ± 0.09	0.56 ± 0.08	0.57 ± 0.10	0.06 ± 0.17
SVM	0.36 ± 0.14	0.50 ± 0.12	0.43 ± 0.10	0.38 ± 0.12	0.47 ± 0.09	0.54 ± 0.12	–0.14 ± 0.21
NN	0.36 ± 0.14	0.38 ± 0.13	0.37 ± 0.10	0.33 ± 0.10	0.40 ± 0.11	0.43 ± 0.11	–0.27 ± 0.19
Ensemble	0.29 ± 0.11	0.38 ± 0.12	0.33 ± 0.09	0.29 ± 0.09	0.38 ± 0.10	0.44 ± 0.11	–0.34 ± 0.18

These values are the mean ± SD that were derived through 10-fold Monte Carlo cross-validation for the classification. SD = standard deviation; Models used to perform the classification: PLS-DA—partial least squares discriminant analysis, RF—random forest, SVM—support vector machine, NN—neural network, Ensemble–integrated model combining predictions from RF, SVM, and NN. PPV—positive predicted value; NPV—negative predicted value; AUC—area under curve; MCC—Matthews correlation coefficient.

**Table 5:** Performance metrics (Mean ± SD) of five classification models for predicting high milk FPR (>1.5) under heat-associated condition (C2).

Model	Sensitivity	Specificity	Accuracy	PPV	NPV	AUC	MCC
PLS-DA	0.10 ± 0.10	1.00 ± 0.00	0.82 ± 0.02	1.00 ± 0.48	0.81 ± 0.02	0.65 ± 0.09	0.29 ± 0.19
RF	0.00 ± 0.00	1.00 ± 0.00	0.80 ± 0.00	0.00 ± 0.00	0.80 ± 0.00	0.69 ± 0.09	0.00 ± 0.00
SVM	0.00 ± 0.00	1.00 ± 0.00	0.80 ± 0.00	0.00 ± 0.00	0.80 ± 0.00	0.59 ± 0.11	0.00 ± 0.00
NN	0.30 ± 0.14	0.95 ± 0.04	0.82 ± 0.04	0.60 ± 0.25	0.84 ± 0.03	0.60 ± 0.11	0.33 ± 0.18
Ensemble	0.10 ± 0.10	0.97 ± 0.03	0.80 ± 0.03	0.50 ± 0.38	0.81 ± 0.02	0.69 ± 0.11	0.15 ± 0.18

These values are the mean ± SD that were derived through 10-fold Monte Carlo cross-validation for the classification. SD = standard deviation; Models used to perform the classification: PLS-DA—partial least squares discriminant analysis, RF—random forest, SVM—support vector machine, NN—neural network, Ensemble—integrated model combining predictions from RF, SVM, and NN. PPV—positive predicted value; NPV—negative predicted value; AUC—area under curve; MCC—Matthews correlation coefficient.

**Table 6:** Performance metrics (Mean ± SD) of five classification models for detecting elevated milk temperature under condition C3 (THI > 70).

Model	Sensitivity	Specificity	Accuracy	PPV	NPV	AUC	MCC
PLS-DA	0.72 ± 0.04	0.70 ± 0.05	0.71 ± 0.02	0.70 ± 0.03	0.72 ± 0.04	0.78 ± 0.03	0.42 ± 0.05
RF	0.88 ± 0.02	0.85 ± 0.03	0.87 ± 0.01	0.86 ± 0.02	0.87 ± 0.02	0.93 ± 0.02	0.75 ± 0.03
SVM	0.75 ± 0.03	0.73 ± 0.04	0.74 ± 0.03	0.74 ± 0.03	0.75 ± 0.03	0.80 ± 0.04	0.48 ± 0.06
NN	0.79 ± 0.03	0.76 ± 0.03	0.78 ± 0.02	0.77 ± 0.03	0.79 ± 0.02	0.85 ± 0.03	0.58 ± 0.04
Ensemble	0.91 ± 0.02	0.88 ± 0.02	0.90 ± 0.01	0.89 ± 0.01	0.90 ± 0.02	0.95 ± 0.01	0.81 ± 0.02

These values are the mean ± SD that were derived through 10-fold Monte Carlo cross-validation for the classification. SD = standard deviation; Models used to perform the classification: PLS-DA—partial least squares discriminant analysis, RF—random forest, SVM—support vector machine, NN—neural network, Ensemble—integrated model combining predictions from RF, SVM, and NN. PPV—positive predicted value; NPV—negative predicted value; AUC—area under curve; MCC—Matthews correlation coefficient.

**Table 7:** Performance metrics (Mean ± SD) of five classification models for detecting low reticulorumen pH under heat stress (Condition C4).

Model	Sensitivity	Specificity	Accuracy	PPV	NPV	AUC	MCC
PLS-DA	0.91 ± 0.07	0.89 ± 0.06	0.90 ± 0.05	0.87 ± 0.06	0.92 ± 0.05	0.97 ± 0.02	0.80 ± 0.09
RF	1.00 ± 0.00	1.00 ± 0.00	1.00 ± 0.00	1.00 ± 0.00	1.00 ± 0.00	1.00 ± 0.00	1.00 ± 0.00
SVM	0.77 ± 0.11	0.93 ± 0.05	0.86 ± 0.05	0.89 ± 0.06	0.83 ± 0.06	0.95 ± 0.03	0.71 ± 0.11
NN	0.86 ± 0.07	0.89 ± 0.06	0.88 ± 0.04	0.86 ± 0.06	0.89 ± 0.05	0.98 ± 0.01	0.75 ± 0.08
Ensemble	0.95 ± 0.05	0.96 ± 0.04	0.96 ± 0.03	0.95 ± 0.04	0.96 ± 0.04	0.99 ± 0.01	0.92 ± 0.05

These values are the mean ± SD that were derived through 10-fold Monte Carlo cross-validation for the classification. SD = standard deviation; Models used to perform the classification: PLS-DA—partial least squares discriminant analysis, RF—random forest, SVM—support vector machine, NN—neural network, Ensemble—integrated model combining predictions from RF, SVM, and NN. PPV—positive predicted value; NPV—negative predicted value; AUC—area under curve; MCC—Matthews correlation coefficient.

**Table 8:** Performance metrics (Mean ± SD) of five classification models for detecting elevated milk FPR (>1.5) under heat stress condition (C5).

Model	Sensitivity	Specificity	Accuracy	PPV	NPV	AUC	MCC
PLS-DA	0.95 ± 0.04	0.98 ± 0.02	0.97 ± 0.02	0.97 ± 0.02	0.96 ± 0.03	0.99 ± 0.00	0.93 ± 0.04
RF	0.97 ± 0.03	1.00 ± 0.00	0.99 ± 0.01	1.00 ± 0.00	0.98 ± 0.02	1.00 ± 0.00	0.98 ± 0.02
SVM	0.82 ± 0.06	1.00 ± 0.00	0.92 ± 0.02	1.00 ± 0.00	0.88 ± 0.03	0.99 ± 0.01	0.85 ± 0.05
NN	0.95 ± 0.04	0.98 ± 0.02	0.97 ± 0.02	0.97 ± 0.02	0.96 ± 0.03	0.999 ± 0.001	0.93 ± 0.04
Ensemble	0.95 ± 0.04	1.00 ± 0.00	0.98 ± 0.02	1.00 ± 0.00	0.96 ± 0.02	0.999 ± 0.001	0.96 ± 0.03

These values are the mean ± SD that were derived through 10-fold Monte Carlo cross-validation for the classification. SD = standard deviation; Models used to perform the classification: PLS-DA—partial least squares discriminant analysis, RF—random forest, SVM—support vector machine, NN—neural network, Ensemble—integrated model combining predictions from RF, SVM, and NN. PPV—positive predicted value; NPV—negative predicted value; AUC—area under curve; MCC—Matthews correlation coefficient.

**Table 9:** Performance metrics (Mean ± SD) of five classification models for detecting combined internal and external heat stress (Condition C6).

Model	Sensitivity	Specificity	Accuracy	PPV	NPV	AUC	MCC
PLS-DA	0.83 ± 0.11	1.00 ± 0.00	0.88 ± 0.08	1.00 ± 0.00	0.71 ± 0.14	0.93 ± 0.06	0.77 ± 0.12
RF	1.00 ± 0.00	1.00 ± 0.00	1.00 ± 0.00	1.00 ± 0.00	1.00 ± 0.00	1.00 ± 0.00	1.00 ± 0.00
SVM	0.83 ± 0.10	0.60 ± 0.24	0.76 ± 0.10	0.83 ± 0.09	0.60 ± 0.21	0.93 ± 0.06	0.43 ± 0.25
NN	1.00 ± 0.00	1.00 ± 0.00	1.00 ± 0.00	1.00 ± 0.00	1.00 ± 0.00	1.00 ± 0.00	1.00 ± 0.00
Ensemble	1.00 ± 0.00	1.00 ± 0.00	1.00 ± 0.00	1.00 ± 0.00	1.00 ± 0.00	1.00 ± 0.00	1.00 ± 0.00

These values are the mean ± SD that were derived through 10-fold Monte Carlo cross-validation for the classification. SD = standard deviation; Models used to perform the classification: PLS-DA—partial least squares discriminant analysis, RF—random forest, SVM—support vector machine, NN—neural network, Ensemble—integrated model combining predictions from RF, SVM, and NN. PPV—positive predicted value; NPV—negative predicted value; AUC—area under curve; MCC—Matthews correlation coefficient.

**Table 10:** Descriptive statistics of the milk and physiological parameters across six heat stress and metabolic threshold conditions in dairy cows.

	Descriptives										
Parameter	Group	N	Mean	Std. Deviation	Std. Error	95% Confidence Interval for Mean		Minimum	Maximum	Cohen’s d	Significant
						Lower Bound	Upper Bound				
Reticulorumen pH	C1	148	6.03	0.28	0.02	5.98	6.07	5.21	6.73	−0.212	0.013
	Control	1312	6.09	0.29	0.01	6.07	6.10	4.94	6.99		
Milk fat-to-protein ratio	C2	243	1.28	0.29	0.02	1.24	1.32	0.69	2.68	0.258	0.0002
	Control	1217	1.20	0.31	0.01	1.19	1.22	0.33	2.86		
Milk temperature (°C)	C3	126	38.77	0.42	0.04	38.69	38.84	37.16	40.26	0.637	<0.00001
	Control	1334	38.52	0.34	0.01	38.50	38.54	37.70	41.15		
Milk lactose (%)	C4	107	4.58	0.39	0.04	4.51	4.66	3.82	5.80	−0.114	0.243
	Control	1353	4.63	0.42	0.01	4.60	4.65	3.03	5.97		
Milk lactose (%)	C5	193	4.60	0.46	0.03	4.54	4.67	3.03	5.88	−0.062	0.443
	Control	1267	4.63	0.42	0.01	4.60	4.65	3.16	5.97		
Rumination time (min/day)	C6	157	424.41	85.03	11.26	402.33	446.48	197.67	575.18	−0.344	0.013
	Control	1403	453.56	84.39	2.25	449.15	457.98	34.67	630.80		

## Data Availability

All pertinent details are contained within the document. All machine learning scripts utilised in this work were authored in Python and may be obtained from the corresponding author upon reasonable request to guarantee transparency and reproducibility of the findings.

## Abbreviations

The following abbreviations are used in this manuscript:

THI	Temperature–Humidity Index
PLS-DA	Partial Least Squares Discriminant Analysis
RF	Random Forest
SVM	Support Vector Machine
NN	Neural Network
SHAP	Shapley Additive Explanations
SCC	Somatic Cell Score
ML	Machine Learning
FPR	Fat-to-Protein Ratio
TMR	Total Mixed Ration
NRC	Nutritional Requirements of Dairy Cattle
RMSEP	Root Mean Square Error of Prediction
A/D	Analog-to-Digital
C1	Condition 1
C2	Condition 2
C3	Condition 3
C4	Condition 4
C5	Condition 5
C6	Condition 6
SARA	Sub-acute Ruminal Acidosis
RBF	Radial Basis Function
MLP	Multi-Layer Perceptron
ROC	Receiver Operating Characteristic Curve
MCC	Matthews Correlation Coefficient
PPV	Positive Predictive Value
NPV	Negative Predictive Value
AUC	Area Under the Curve
ANOVA	One-Way Analysis of Variance
SD	Standard Deviations
SEM	Standard Errors of the Mean
CI	Confidence Intervals
PLF	Precision Livestock Farming
ANN	Artificial Neural Networks
KNNR	K Nearest Neighbours for Regression
DTR	Decision Tree for Regression
LSTM	Long Short-Term Memory
FTIR	Fourier-Transform Infrared
VFA	Volatile Fatty Acids
HCO_3_	Bicarbonate
CO_2_	Carbon Dioxide
SDA	Stepwise Discriminant Analysis
CDA	Canonical Discriminant Analysis
DA	Discriminant Analysis
CNN	Convolutional Neural Network
pCO_2_	Carbon Dioxide Pressure
pO_2_	Partial Oxygen Pressure
VIF	Variance Inflation Factor
